# Biomarkers to predict the onset of biphosphonate-related osteonecrosis of the jaw: A systematic review

**DOI:** 10.4317/medoral.22763

**Published:** 2019-01

**Authors:** Alejandro I. Lorenzo-Pouso, Mario Pérez-Sayáns, Sergio González-Palanca, Cintia Chamorro-Petronacci, José Bagán, Abel García-García

**Affiliations:** 1 DDS, MSc. Oral Medicine, Oral Surgery and Implantology Unit, Faculty of Medicine and Odontology, University of Santiago de Compostela, Santiago de Compostela, Spain. GI-1319 Research Group, Health Research Institute of Santiago de Compostela (IDIS), Santiago de Compostela, Spain; 2 DDS, PhD Oral Medicine, Oral Surgery and Implantology Unit, Faculty of Medicine and Odontology, University of Santiago de Compostela, Santiago de Compostela, Spain. GI-1319 Research Group, Health Research Institute of Santiago de Compostela (IDIS), Santiago de Compostela, Spain; 3 MD, PhD. Department of Gynecology & Obstetrics, Valdeorras Hospital, El Barco de Valdeorras, Spain; 4 DDS, MSc, PhD. Oral Medicine, Oral Surgery and Implantology Unit, Faculty of Medicine and Odontology, University of Santiago de Compostela, Santiago de Compostela, Spain. GI-1319 Research Group, Health Research Institute of Santiago de Compostela (IDIS), Santiago de Compostela, Spain; 5 MD, DDS, PhD. Oral Medicine, University of Valencia, Valencia, Spain; Service of Stomatology and Maxillofacial Surgery, University General Hospital, Valencia, Spain; 6 MD, PhD. Oral Medicine, Oral Surgery and Implantology Unit, Faculty of Medicine and Odontology, University of Santiago de Compostela, Santiago de Compostela, Spain. GI-1319 Research Group, Health Research Institute of Santiago de Compostela (IDIS), Santiago de Compostela, Spain

## Abstract

**Background:**

The goal of this paper was to identify available biomarkers to predict the onset of biphosphonate-related osteonecrosis of the jaw (BRONJ).

**Material and Methods:**

Case-control studies comparing the different concentrations of a series of molecules detected in serum and urine as matrices of BRONJ affected patients vs. non-affected were included. PRISMA guidelines for systematic reviews were used for the present paper. Two reviewers independently screened electronic databases (Medline, Web of science, and The Cochrane Library) and performed hand searches. Risk of bias assessment of selected studies was performed by the Newcastle-Ottawa Scale. This study is registered as PROSPERO CRD42017078149.

**Results:**

From a total of 601 identified studies, 7 (4 articles with high methodological quality and 3 with medium) articles were included. They investigate 2623 patients, of whom 91 (3.47%) developed BRONJ. A total of 7 biomarkers were identified and classified into 3 groups: bone turnover, angiogenesis and endocrine markers. Conflicting results were found in relation to most biomarkers.

**Conclusions:**

The present review suggests that no useful markers are currently available to evaluate BRONJ risk. Nevertheless, the present paper indicates that a paradigm shift from bone turnover biomarkers to angiogenesis and endocrine markers could shed light on this search.

** Key words:**Biphosphonate, jaw, osteonecrosis, osteoporosis.

## Introduction

Bisphosphonates (BPs) are analogues of pyrophosphate which have potent inhibitory effects on bone resorption (1,2). These bone targeted-therapies are widely used for osteoporosis and different types of cancers. BPs have been proven to reduce the risk of skeletal-related events (SREs), delay the onset of SREs and ease bone-related pain (3).

There are two main classes of BPs from a chemical standpoint: non-nitrogen containing bisphosphonates (i.e. etidronate and clodronate) and nitrogen containing bisphosphonates (nBPs); this last group is subdivided into the alkyl-amino bisphosphonates (i.e. pamidronate, alendronate and ibandronate) and the heterocyclic nitrogen containing bisphosphonates (i.e. risendronate and zolendronate) (4,5).

Non-nitrogen containing bisphosphonates are metabolised in the cells to an adenosine triphosphate derivative that impairs osteoclast function and induces osteoclastic apoptosis. In the case of nBPs, the bone-targeting pharmacokinetic properties of these drugs cause selective inhibition of farnesyl diphosphatase synthase and a reduction in the production of prenylated forms of guanosine thiphosphate biding proteins (GTPases) causing an inhibition of osteoclast activity and generating an increase in bone turnover and bone mineral density (BMD) (6). nBPs have a higher relative potency in relation to non-nitrogen containing bisphosphonates. Alkyl-amino bisphosphonates are 100 ~1000 times more potent that non-nitrogen containing bisphosphonates. Third-generation BPs also known as heterocyclic nitrogen containing bisphosphonates have the highest relative potency of all BPs, they are 1000 ~20000 times more potent that etidronate (1,4).

Bisphosphonate-related osteonecrosis of the jaw (BRONJ) is infrequent sequelae of these types of antiresorptive drugs (7). According to the American Association of Oral and Maxillofacial Surgeons (AAOMS), BRONJ is defined as an area of exposed bone in the maxillofacial region in a patient treated with bisphosphonates (BPs) and who did not receive radiotherapy in the craniofacial region that does not heal during the 8 weeks following its identification by a health care provider (8). The physiopathology of this outcome remains partially unknown; however, currently there are three main theories to explain its aetiology: 1) inhibition of osteoclast activity with a suppression of bone turnover, 2) the relationship of inflammation with infection, and 3) inhibition of angiogenesis (9,10). These explanations arise from the current state-of-the-art in bone biology.

The maxillary and mandible bones concentrate a greater proportion of BPs than other bone tissues due to their relatively higher bone turnover ratio (11). This remodelling rates cause an alveolar bone cortical thickness (12). In addition, to this osteoclastic activity suppression, BPs can also suppress angiogenesis through an inhibitory effect on vascular endothelial cells (13). Furthermore, the presence of microflora distinct to the oral cavity has been recently considered as a potential trigger or stimulating factor in the progress of this outcome; in this sense, BRONJ-related bone sequestra have shown the presence of bacteria such as Fusobacterium, Eikenella, Bacillus, Actinomyces, Staphylococcus and Streptococcus (14). This biofilm causes an increase of circulating cytokines and a dysfunction of matrix proteases which leads to a chronic inflammatory response (13). Impaired macrophage function following bacterial stimulation may be linked to this outcome. Since osteoclasts and macrophages have the same cell lineage (i.e. granulocyte/monocyte progenitor); it’s plausible that BPs inhibit the activity of macrophages and also the differentiation from macrophage/monocyte to osteoclasts (15).

On the other hand, at the systemic and oral levels, there are fundamental risk factors involving the aetiology of the entity that must be understood. At the oral level, the risk factors include dental extraction and periodontal disease. At a systemic level, different types of treatments, such as chemotherapy, treatment with corticosteroids and anti-angiogenic therapies, are risk factors associated with BRONJ. Other factors related to the development of BRONJ are closely related to BPs, such as the type of medication, route of administration and duration of treatment (10). In addition, certain genetic predispositions have also been described, including CYP2C8 gene polymorphisms, vascular endothelial growth factor gene polymorphisms, and mutations in the prothrombin gene (16). Recently, other drugs have been linked to osteonecrosis of the jaw onset, such as inhibitors of nuclear factor-kB ligand (RANKL), angiogenesis inhibitors, tyrosine kinase receptors and tumour necrosis factor alpha (TNF-α) have been identified, motivating the name change to ‘medication-related osteonecrosis of the jaws’ (MRONJ) (17).

In 2003, Marx described the first case series of BRONJ-affected patients (18). In 2007, this same author proposed carboxy-terminal collagen crosslinks in serum (sCTX) as a biomarker to predict the risk of recurrence of this unwanted complication in a patient undergoing antiresorptive treatment. The data suggested that a CTX value below 100 pg/ml represents a high risk of BRONJ relapse, values between 100 and 150 pg/ml represent a moderate risk, and values greater than 150 pg/ml represent a minimal risk (19). This biomarker was later proved not to be predictive. In fact, a recent meta-analysis confirms its inefficiency in this regard (20). The main reason for the dysfunction of this biomarker according to the American Society for Bone and Mineral Research (ASBMR) task force is that low sCTX levels are simply a reflection of the pharmacological effects of antiresorptive therapies (21). The positioning of the AAOMS in 2014 confirms that non-predictive biomarkers are available for this pathological entity (8). Since then, different researchers worldwide have focused their efforts on identifying useful markers for this pathology.

As observed, there is a lack of consensus in the literature regarding the use of biomarkers to predict the onset of BRONJ; both the AAOMS (8) and the ASBMR (7) agree on that there is insufficient evidence to prove the efficacy of any biomarker in the BRONJ prevention protocols. To our best knowledge no previous systematic approach to this emerging topic can be found in the literature.

In the present review, the most relevant advances achieved to date in the search for a biomarker to assess the risk of developing BRONJ are comprehensively compiled and discussed. We will focus on serum and urine markers, describing only briefly some investigations on alternative matrices. We will only review research that seeks to establish the risk of disease prior to development of said disease, not biomarkers that evaluate prognosis or staging.

## Material and Methods

-Search protocol

The protocol of this systematic review was designed by AL-P and registered in PROSPERO (CRD42017078149). This review was conducted based on the PRISMA (Preferred Reporting Items for Systematic Reviews and Meta-analysis) guidelines (22).

The review used the PICO methodology: adults (P = patients); development of BRONJ; (I = intervention), no development of BRONJ (C = comparison); correlation with a biomarker (O = outcome. The objective was to answer the following question: What are the most effective biomarkers for the risk assessment of developing BRONJ?

-Eligibility criteria

To select the studies included in this systematic review, the publications had to meet the following criteria: 1) prospective or retrospective case-control studies; 2) performed on humans older than 18 years; 3) research evaluates biomarkers to predict the presence of mandibular osteonecrosis in patients treated with bisphosphonates; 4) data on the type of BP, duration of treatment and route of administration must be provided; and 5) the diagnostic criteria of BRONJ must be valid (7,8).

Criteria for exclusion were as follows: 1) contained less than 10 patients; 2) included patients under 18 who consumed BPs due to osteogenesis imperfecta; 3) written in a language other than English; 4) publications with redundant material; 5) animal studies; 6) included patients treated with radiotherapy in the maxillofacial region; and 7) dealt with other entities similar to BRONJ (e.g., oral ulceration and bone sequestration (OUBS)) or whose origin is attributed to other drugs.

-Systematic search

A review was made using the MEDLINE databases (via PubMed, from September 2003 to September 2017), Web of Science (WOS) (from September 2003 to September 2017) and The Cochrane Library (CL) (from September 2003 to September 2017). This search was performed during October 2017. A manual search was also performed in a series of journals: Bone; British Journal of Oral and Maxillofacial Surgery; International Journal of Oral and Maxillofacial Surgery; Journal of Oral Maxillofacial Surgery; Journal of Cranio-Maxillofacial Surgery; Oral Surgery, Oral Medicine Oral Pathology and Oral Radiology and Oral Oncology. Potentially relevant articles that were known to any of the review authors and reference lists of retrieved articles were also checked.

The search in PubMed via Medline was based on the following terms: ((diphosphonates OR bisphosphonates OR antiresorptive) AND (osteonecrosis OR jaw osteonecrosis) AND (biomarkers OR biological markers)). The search used both MeSH terms and free search. This search was conveniently adapted for use in WOS and CL.

-Data extraction and analysis 

Data extraction and collection were performed by a group of research experts in the field of oral surgery (AL-P and MP-S) and a maxillofacial surgeon (AG-G) according to the previously described criteria. All abstracts and research titles that emerged from the initial search were analysed. Once that first screening was performed, the full texts of selected papers were analysed. The reason for exclusion was recorded for those items that were eliminated in this phase. Consensus between the main researchers (AL-P and MP-S) was acceptable during the inclusion process. The agreement in this process was calculated using Cohen’s kappa coefficient, and a κ score of 0.8 was obtained. In case of discrepancy, the third researcher (AG-G) acted as a mediator.

The following data were extracted and analysed in all the studies included in the review: sample size, criteria for the diagnosis of BRONJ, type of BP, route of administration, underlying disease, covariate adjustment, biomarker (matrix) and main results.

-Risk of bias evaluation

The methodological quality of the included studies and the possibility of bias were assessed using the Newcastle-Ottawa Scale (NOS) (23). This scale measures three dimensions (selection, comparability of cohorts, and outcome) with a total of 9 items. In the analysis, the studies with NOS scores of 1-3, 4-6 and 7-9 were defined as low, medium and high quality, respectively. This analysis was executed for each study independently by AL-P and MP-S. In the case of a disagreement between the two researchers, a third researcher (AG-G) acted as mediator.

Simultaneously, an individual assessment of each item was obtained regarding the level of evidence (LoE) according to the Oxford CEBM Levels of Evidence classification (24).

## Results

**Figure 1:**
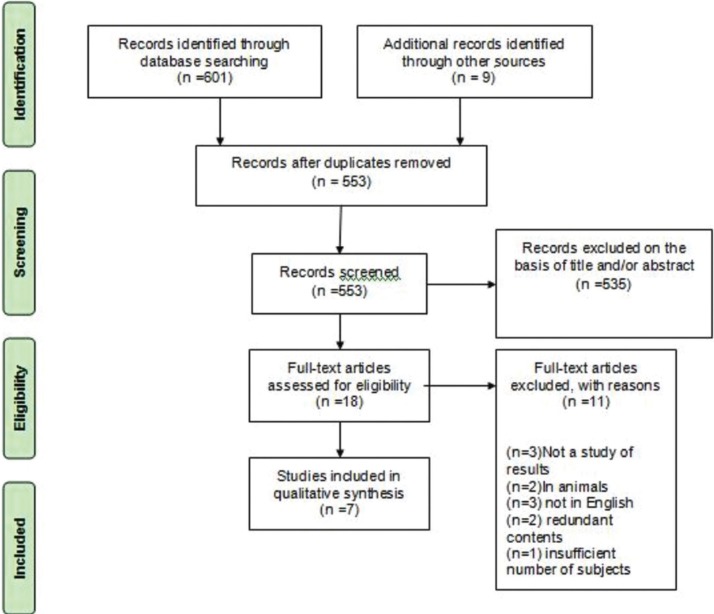
Flow diagram of the systematic review.

The search process was reduced to 601 titles and abstracts that were subjected to evaluation after eliminating 48 duplicate publications. After reading the abstracts, 535 articles were eliminated because they did not meet the criteria. After examining the full text, 11 documents were excluded for different reasons. Therefore, 7 articles were included in the present systematic review (Fig. 1).

A summary of the 7 selected articles (25-31) and their main results can be found in Table 1, 1 continue. The studies are organized chronologically from the oldest to the most recent publication.

These works were performed in 6 different countries on four continents (Europe, Asia, America and Oceania). All studies were published between 2009 and 2017. The sample sizes of the included works varied from 50 subjects in the study by Topaglu *et al.* (31) to 1900 subjects in the study by Hutchenson *et al.* (30). In total, the review includes 2623 patients, of whom only 91 (3.47%) developed BRONJ. In relation to the diagnosis of BRONJ, the most widely used criteria were those of AAOMS (8), used in 4/7 of the studies ( Table 1,  Table 1 continue). The global distribution regarding the types of BPs used by the patients was as follows: 812 alendronate (57.02%), 8 clodronate (0.56%), 2 etidronate (0.14%), 16 ibandronate (1.12%), 7 pamidronate (0.49%), 469 risedronate (32.77%), 100 zoledronate (6.99%) and 13 other combinations (0.91%). The following underlying diseases were noted in the patients: 1361 osteoporosis (91.77%), 40 breast cancer (2.70%), 31 multiple myeloma (2.09%), 19 bone metastases (1.28%), 11 prostate cancer (0.74%), 8 lung cancer (0.54%), 5 Paget’s disease (0.34%), 3 kidney cancer (0.20%), 2 nasopharyngeal cancer (0.13%), 2 thyroid cancer (0.13) and 1 neurogenic cancer (0.07%). Three included articles (25,28,29) did not specify the primary malignant diseases responsible for bone metastases.

**Table 1:**
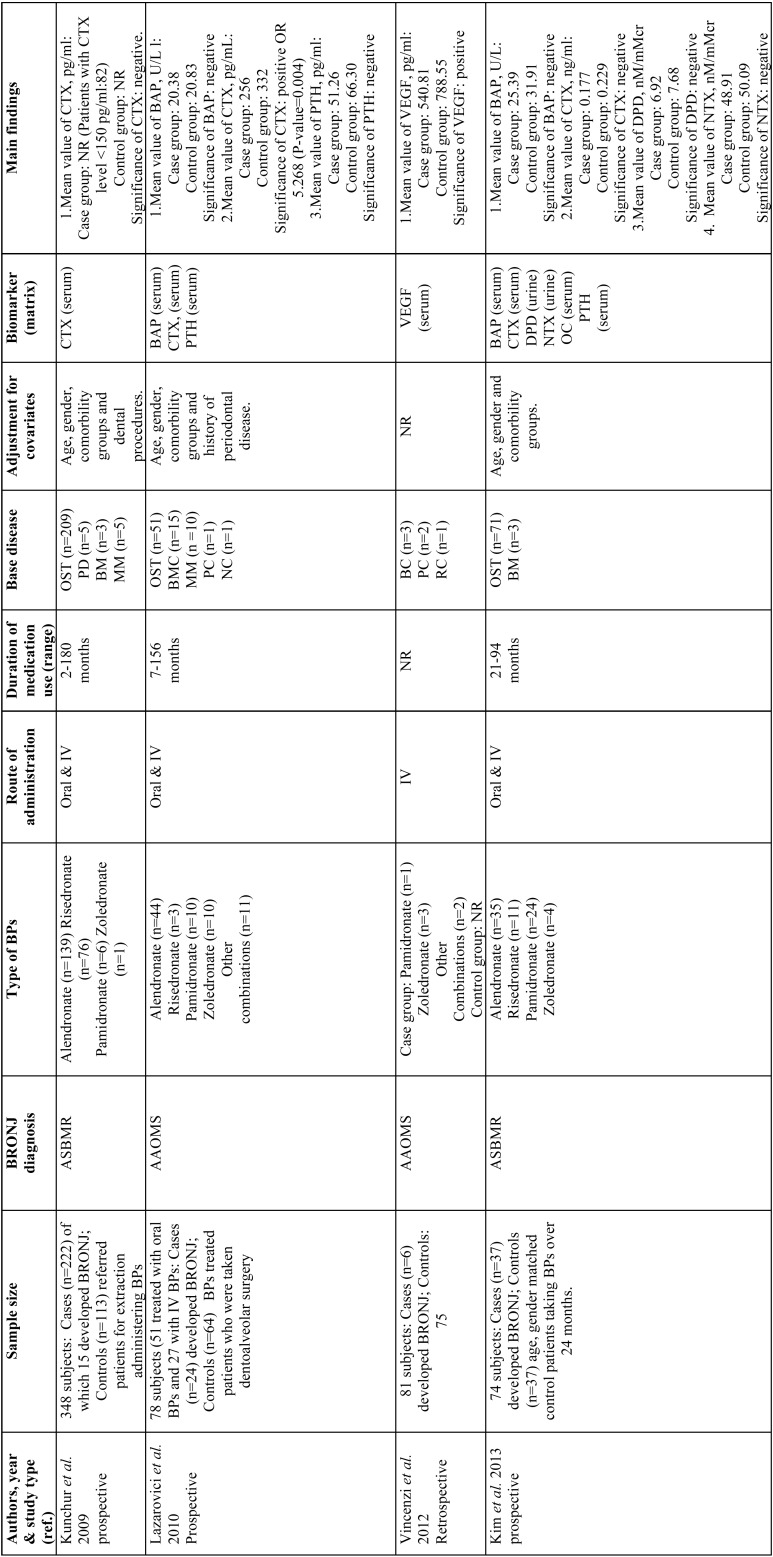
The main characteristics of the included controlled Studies.

**Table 1 continue:**
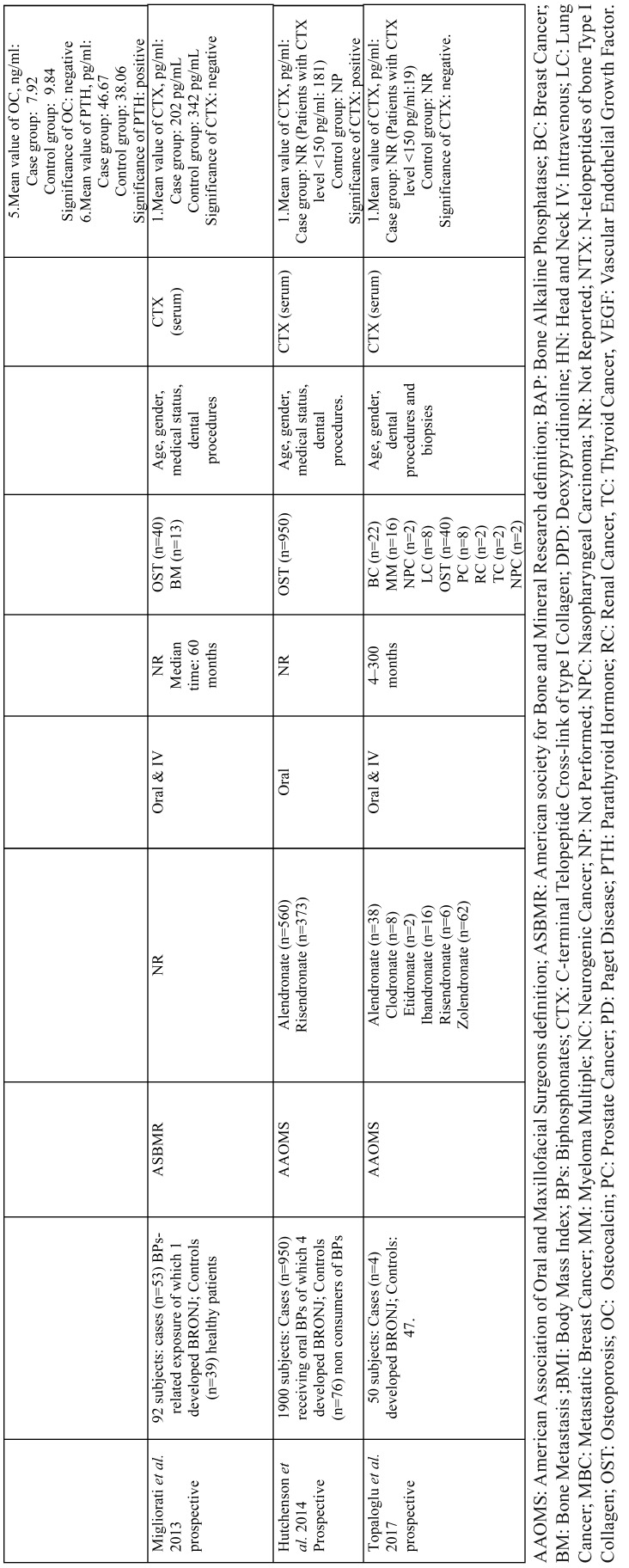
The main characteristics of the included controlled Studies.

A total of 7 biomarkers were identified and classified into three groups: bone turnover biomarkers (i.e., bone alkaline phosphatase (BAP), c-terminal telopeptide cross-link of type I collagen (CTX), deoxypyridinoline (DPD), N-telopeptides of bone type I collagen (NTX), osteocalcin (OC)), endocrine biomarkers (i.e., parathyroid hormone (PTH)), and angiogenesis markers (i.e., vascular endothelial growth factor (VEGF)).

The marker most frequently used was CTX, used in 6 of the 7 investigations; this marker was only reported as a predictor of BRONJ in two studies (26,30). VEGF was also shown to be predictive in a single study (27). PTH was predictive in one study of the two studies that evaluated it (28). On the other hand, BAP, DPD, NTX and OC were not effective predictors in any study ( Table 1) (26,28).

**Figure 2:**
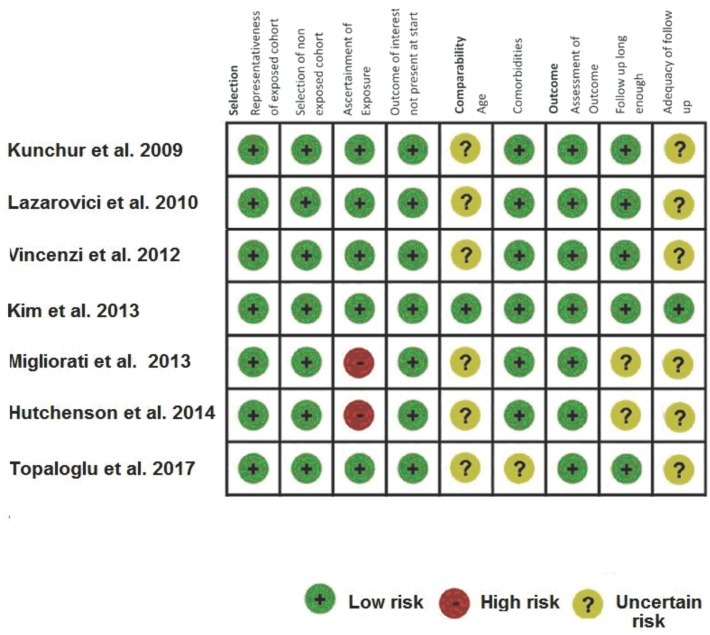
Risk of bias assessment according to NOS (16).

Regarding the evaluation of the risk of bias according to NOS (23), 4 articles with high methodological quality were identified with NOS scores between 7-9 (25,28), and 3 articles were of medium quality with scores between 4-6 (29-31) (Fig. 2). The level of evidence of all the works included according to the aforementioned classification was IIIb (24).

## Discussion

The search for useful predictive markers to assess the risk of BRONJ onset has proved to be extremely complex due to its low incidence, estimated at 0.7 per 100,000 people per year (8). Evidence of this low incidence is the small number of documents included in this review. On the other hand, numerous papers evaluate these biomarkers after the pathology has already occurred in an attempt to assess the risk of a relapse after a ¨drug holiday¨ or to identify markers that correlate with disease stage (32-35). Not all drug holidays are useful in preventing this pathology because based on their pharmacokinetic nature, BPs irreversibly accumulate in the bone in a very short period of time (8). The total prevalence of BRONJ in the present review was 3.47% of the total number of patients. This value is within the range of previous epidemiological studies in similar communities, 0.01-12%. It’s important to highlight that prevalence range from under 1% for patients with primary osteoporosis and up to about 20% for high-risk subpopulations (i.e. patients with a malignant disease and further local or systemic risk factors) (17).

The BPs most frequently used by the patients in this review were alendronate and risedronate (98.79%), which are considered first choice antiresorptive drugs in osteoporotic patients. The third most used BP was zoledronate (6.99%), which is a good option for osteoporosis in cases of digestive intolerance, poor adherence to treatment or increased risk of bone fractures. For many oncologic diseases (i.e. breast cancer, multiple myeloma or prostate cancer) zoledronate has proven to be the drug of first choice. In case of renal failure, patients are more likely to experience renal impairment with zoledronate, which is why in these situations it is advisable to use ibandronate (36).

It should be noted that Marx *et al.* suggested the use of CTX as a biomarker of the risk of relapse (19). To the best of our knowledge, the first work to assess the utility of CTX as a biomarker of initial risk was carried out by Kunchur *et al.* (25). This distinction in the search for markers to predict this pathology has not been addressed to date. However, recently, McGowan *et al.* postulated a list of potential serum biomarkers (i.e., VEGF, ESR, CRP and CTX) (37). We classified the list of markers produced in this systematic review into three groups: bone turnover biomarkers, endocrine biomarkers and angiogenesis markers. These markers were nominated by the current BRONJ aetiopathogenic model at the time and given their intimate relationship with osteoporosis (6).

In the case of osteoporosis, bone turnover biomarkers present a series of individual limitations that prevent osteoporosis from being useful for diagnosis (6,38). In fact, its basic utility in medicine is to advise the response to treatment. Some inherent limitations of these biomarkers are highlighted below. BAP has a low sensitivity and specificity in the study of bone metabolic disease and is not useful in patients with hepatic disorders. Osteocalcin appears altered in states of liver failure. CTX and NTX do not exclusively measure bone metabolism but all the tissues that contain type I collagen. Finally, DPD is currently considered a non-discriminatory marker in bone pathology. In the study of osteoporosis, the current gold standard biomarker is CTX, which has a good correlation with BMD. It’s important to consider that bone turnover biomarkers fall within wide limits. This factor jeopardizes the validity of strategic planning through biomarker sampling in relation to this outcome (39).

Specifically, regarding BRONJ, this score is not useful according to the results of this review despite the significant heterogeneity of the data. The recent systematic reviews of Dal Prá *et al.* (40) and Enciso *et al.* (20) reached the same conclusion. An important limitation is the significant divergence between the control groups, among which healthy subjects (29,30), patients treated with BPs (27) or patients who consume BPs undergoing dentoalveolar surgery are included (25,26,28,31). In total, 91.7% of the patients included in this review suffered from osteoporosis as an underlying disease. This finding is consistent with a recent review in which it was also the most frequent pathology (40). The lack of correlation between these biomarkers and the development of BRONJ indicates that the correct therapeutic control of the underlying disease does not seem to play a key role in the prevention of BRONJ. Studies that evaluated these biomarkers in patients who stopped consuming BPs and in controls who continued with these treatments also did not find these biomarkers useful (35). Kim *et al.* (28) performed an analysis of ROC curves for some of these biomarkers, in which CTX reached a sensitivity of 29.73% and a specificity of 89.19% with a cut-off value of ≤ 0.094 ng/ml. However, Hutcheson concluded that a CTX value <150 pg/ml at the time of tooth extraction is associated with a 3-fold increased risk of developing BRONJ (30). In our opinion, the use of these markers is not justified for assessing the risk for developing BRONJ, and scientific evidence is not available to support their use in a therapeutic protocol. However, caution should be taken when assessing this statement due to the limited number of BRONJ cases or the within-group heterogeneity in the control groups.

It has been postulated that the inhibition of angiogenesis can play a key role in the development of BRONJ (9,41). This relationship is due to the role that osteoclasts play in angiogenesis. Osteoclasts are actively involved in the production of blood vessels through the production of matrix metalloproteinase-9 (MMP-9). Zoledronate and pamidronate inhibit the production of MMP-9 and subsequently angiogenesis (42). Taking into account the higher concentration of BPs that occurs in the maxillary bones, it seems reasonable that this effect plays a key role in the development of this pathology (43). The biomarker that was proposed in this study was VEGF. VEGF is a fundamental protein in vasculogenesis and angiogenesis and therefore considered an important biomarker in some autoimmune pathologies and tumours. Its outstanding role in relation to BRONJ is demonstrated by the described cases of osteonecrosis of the jaw induced by bevacizumab, a monoclonal antibody that acting on VEGF inhibits angiogenesis (17). In the only series of patients in this review in which VEGF was used as a biomarker, it was found to be predictive (27). Later, Thumbigere-Math *et al.* demonstrated that it was useful as a biomarker in patients who suffered BRONJ after a long discontinuation of the treatment (35). The evidence regarding this biomarker is limited, and the development of new research is needed to assess its inclusion in the risk protocols. Despite this notion, VEGF seems to have a promising future. A recent study of salivary proteomics in patients with BRONJ also demonstrated significantly increased levels of MMP-9 with respect to patients treated with BPs who did not develop this pathology (44). A body of evidence has established that expression or activity of metalloproteinases (MMPs) may be stimulated by periodontal diseases; these mechanisms could in part explain the high comorbidity of periodontal disease and BRONJ (45).

The last group of biomarkers treated in this review is the endocrine biomarkers. The possible relationship between the appearance of BRONJ after a period of hypocalcaemia and secondary hyperparathyroidism has been proposed (46). Evidence suggests that PTH plays a key role in angiogenesis given that treatment with parathyroid hormone related protein (PTHrP) *in vivo* increases the number of blood vessels and the number of osteoclasts (42). Recently, a novel treatment for BRONJ has been reported based on the use of teriparatide, an analogue of human parathyroid hormone, whose future looks promising in the treatment of BRONJ despite the limited existing evidence (17). The results of the present review are conflicting in this sense. On one hand, the series by Kim *et al.* highlights PTH as a useful biomarker in an analysis using ROC curves with a cut-off value of >41.52 pg/ml, a sensitivity of 56.52% and a specificity of 86.67% (28). In contrast, in the series by Lazarovici *et al.*, this biomarker was not predictive (26). More research is needed to determine whether this biomarker is useful. Other biomarkers related to the endocrine system, such as triiodothyronine (T3) and thyroid stimulating hormone (TSH), have been used to evaluate the risk BRONJ relapse after a long discontinuation of BP consumption; however, these biomarkers were not effective (35).

Some investigations have suggested the use of other biomarkers that have not yet been used predictively, including C-reactive protein (CRP), erythrocyte sedimentation rate (ESR), interleukin-17 (IL-17), α-CTX, tartrate-resistant acid phosphatase 5b (TRACP 5b), receptor activator for nuclear factor κ B ligand (RANKL), and osteoprotegerin (OPG) (21,28,34,35). The most commonly used matrices in search of these biomarkers are serum and urine. Recently, saliva has also been used in the search for biomarkers (44,47-49). In this regard, a series of proteins whose levels are altered in BRONJ patients’ saliva have been detected; mainly molecules in relation to oxidative stress (i.e. glutathione, malondialdehyde, oxidized glutathione, and 8-oxo-7,8-dihydro-2-deoxyguanosine) (47), interleukins (i.e. Interleukin-1 alpha, interleukin-1 receptor antagonist, interleukin 1 beta, and interleukin-6) (48,49) and other proteins (desmoplakin, metalloproteinase-9, mammaglobin-B, carbonic anhydrase II, etc) (44).

The present systematic review has certain limitations. The results are affected by the great variety of health and disease states of the participating subjects, the variety of drugs, administration routes, treatment times, different covariate adjustment, and the different definitions of BRONJ applied. It should be noted that the search for an adequate protocol to predict the risk of suffering from BRONJ or simply provide information regarding its management, regardless of the search for markers, appears to still be in the distant future.

Although the current evidence regarding the use of biomarkers to assess the risk of suffering from BRONJ is limited, this review suggests that no useful markers are currently available to evaluate this risk. However, this review indicates that the paradigm shift from bone turnover biomarkers to angiogenesis and endocrine markers could shed light on this search.

There is a need for the execution of new prospective studies that are capable of collecting representative samples of this infrequent pathology. International cooperation for the creation of new multicentre studies is likely the best option.
